# Draft genome sequence of *Enterobacter cloacae* strain TG-C5.2 isolated from shrimp aquaculture water in the Mekong Delta, Vietnam

**DOI:** 10.1128/mra.00528-26

**Published:** 2026-06-18

**Authors:** Lat Hong Luu, Nhan Thanh Lam, Nguyet Nhu Phan, Trang Thi Phuong Phan

**Affiliations:** 1 Center for Bioscience and Biotechnology, University of Science, Vietnam National University Ho Chi Minh City 54800, Ho Chi Minh City, Vietnam; 2 Vietnam National University 54800, Ho Chi Minh City, Vietnam; 3 National Hospital of Odonto-Stomatology 835576, Ho Chi Minh City, Vietnam; 4 Institute of Public Health, Ho Chi Minh City, Vietnam; Rochester Institute of Technology, Rochester, New York, USA

**Keywords:** *Enterobacter cloacae*, shrimp aquaculture, antimicrobial resistance, blaCTX-M-15

## Abstract

We report the draft genome sequence of *Enterobacter cloacae* TG-C5.2 isolated from shrimp aquaculture water in Vietnam. The genome harbors multiple antimicrobial resistance genes, including *blaCTX-M-15*.

## ANNOUNCEMENT

*Enterobacter cloacae* is an opportunistic gram-negative bacterium commonly associated with antimicrobial resistance in both environmental and clinical settings (1, 2). Here, we report the draft genome sequence of strain TG-C5.2 isolated from shrimp aquaculture water in the Mekong Delta, Vietnam. Shrimp aquaculture environments are considered potential reservoirs for antimicrobial-resistant bacteria due to the frequent use of antibiotics and environmental exposure to antimicrobial residues. Therefore, characterization of environmental *Enterobacter cloacae* isolates may provide useful insights into the dissemination of antimicrobial resistance genes in aquaculture systems.

Strain TG-C5.2 exhibited an extended-spectrum β-lactamase (ESBL) phenotype and high-level resistance to amoxicillin (MIC = 2,048 µg/mL). The isolate was preliminarily identified by matrix-assisted laser desorption/ionization time-of-flight mass spectrometry, and species identity was confirmed by genome-based analysis. National Center for Biotechnology Information (NCBI) quality assessment returned a species_match for *Enterobacter cloacae*, and average nucleotide identity analysis showed 98.96% identity to the closest type-strain assembly.

Water samples were collected from a shrimp aquaculture pond in the Mekong Delta, Vietnam. Samples were filtered through a 0.45 µm membrane filter and cultured on nutrient agar supplemented with cefotaxime (1 mg/L) at 37°C for 24 h. A representative colony was selected and purified prior to further analysis.

Genomic DNA was extracted using the DNeasy Blood and Tissue Kit (Qiagen, Germany). Sequencing libraries were prepared using the TruSeq Nano DNA Library Preparation Kit (Illumina, USA) by Macrogen Inc. (Seoul, South Korea). Whole-genome sequencing was performed on the Illumina NovaSeq 6000 platform using 2 × 150 bp paired-end reads, generating approximately 8.7 million reads with an average coverage of 85×. Raw reads were quality checked using FastQC v0.12.1 (3) and processed using SeqKit v2.8.2 (4). Unless otherwise specified, default parameters were used for all bioinformatic analyses.

*De novo* assembly was carried out using Unicycler v0.5.1 (5) and evaluated with QUAST v5.2.0 (6). Initial genome annotation was performed using Bakta v1.9.4 (7) prior to submission. Following submission to NCBI, the assembly was processed and annotated using the NCBI Prokaryotic Genome Annotation Pipeline (PGAP), and the PGAP/RefSeq annotation was used for reporting the final genome features in this study.

The final RefSeq assembly (GCF_050209095.1) has a genome size of approximately 5.1 Mb and consists of 55 contigs, with a contig N50 of 235 kb and a GC content of 54.5%. PGAP annotation identified 4,873 genes, including 4,729 protein-coding genes. Although the assembly remained fragmented into 55 contigs, the sequencing coverage and assembly statistics were considered sufficient for genome-based characterization of antimicrobial resistance determinants.

Antimicrobial resistance genes were detected using AMRFinderPlus v3.12.8 (8), including *blaCTX-M-15*, *qnrS1*, and the *oqxAB* efflux system. Mobile genetic elements were predicted using ISEScan v1.7.2.3 (9), mobileOG-db v1.6.0 (10), and geNomad v1.8.0 (11), indicating that some resistance determinants may be associated with mobile genetic elements.

The presence of ESBL-associated genes and mobile genetic elements suggests the potential dissemination of antimicrobial resistance determinants in aquaculture-associated environments.

## Data Availability

The draft genome sequence of *E. cloacae* TG-C5.2 has been deposited in DDBJ/ENA/GenBank under accession number JBNPHX000000000. The version described in this paper is the first version, JBNPHX010000000. Raw sequencing reads are available in the Sequence Read Archive under accession number SRR38067203 associated with BioProject PRJNA1233926 and BioSample SAMN47284024.
